# Supplementary material for: Built-In Packaging for Single Terminal Devices
Source: Sensors (Basel). 2022 Jul 14;22(14):5264. doi: 10.3390/s22145264 (PMC9318481; doi:10.3390/s22145264)
Supplement: Supplementary file 1 [file sensors-22-05264-s001.zip › Supplementary Materials.pdf]

Supplementary Materials

Built-in Packaging for Single Terminal Devices

Ahmet Gulsaran [1,2,*], Bersu Bastug Azer [1,2], Samed Kocer [2,3], Sasan Rahmanian [2,3], Resul Saritas [2,3], Eihab M. Abdel-Rahman [2,3] and Mustafa Yavuz [1,2]

[1] Mechanical and Mechatronics Engineering Department, University of Waterloo, Waterloo, ON N2L 3G1, Canada; bbastuga@uwaterloo.ca (B.B.A); myavuz@uwaterloo.ca (M.Y.)

[2] Waterloo Institute of Nanotechnology (WIN), University of Waterloo, Waterloo, ON N2L 3G1, Canada

[3] Systems Design Engineering Department, University of Waterloo, Waterloo, ON N2L 3G1, Canada; skocer@uwaterloo.ca (S.K.), s223rah@uwaterloo.ca (S.R.), resulsaritas@gmail.com (R.S.), eihab@uwaterloo.ca (E.M.A.)

*Correspondence: agulsaran@uwaterloo.ca

Content of Supplementary Materials

1. Table of experimental equipment
2. ROM calculations
    2.1. Mathematical model
        2.1.1. Static analysis
        2.1.2. Dynamic analysis
        2.1.3. Stress analysis
3. SEM images without color
4. Frequency sweep calculations
5. Mode shape videos

1. Table of experimental equipment

Table S1. Equipment list.

Equipment	Brand	Model
Function Generator	BK Precision	4054
Voltage Amplifier	Tabor Electronics	9200
LDV	Polytec	Custom
Oscilloscope	Keysight	DSOS204A

2. ROM calculations

2.1. Mathematical model

In this section, the impact of residual axial stress on the natural frequencies of the clamped-clamped beam is investigated through numerical simulation. To do this, the motion's equation governing the dynamic behavior of the structure, which is prevalently found in the literature [45], is presented in Eq. (1).

$$\rho A\frac{\partial^2 w}{\partial t^2}+EI\frac{\partial^4 w(x)}{\partial x^4}-\left[N+\frac{EA}{2L}\int_0^L\left(\frac{\partial w}{\partial x}\right)^2dx\right]\frac{\partial^2 w}{\partial x^2}=\frac{\epsilon_0 b\left(V_{DC}+V_s(t)\right)^2}{2(d-w)^2} \tag{1}$$

subject to the following boundary conditions,

$$w(0;t)=w'(0;t)=0\ ,\ w(L;t)=w'(L;t)=0 \tag{2}$$

where, ρ, E, L, A, and I are the material density, elastic Young's modulus, beam length, area of the beam cross-section, and the second moment of area of the beam cross-section, respectively. Moreover, b represents the beam width, d is the initial gap distance, and ϵ_0 refers to the permittivity of air filling the gap zone between the moving and stationary electrodes. $w(x;t)$ defines the displacement component of a material particle placed on the beam neutral axis, which is located at the distance of x from the left support. N denotes the axial load that can be either compressive or tensile, creating a so-called membrane stiffness for the transverse vibration. The integral term describes the mid-plane stretching which is caused by fixed boundaries. Defining the following nondimensional parameters,

$$\widetilde{w}=\frac{w}{d}\quad,\quad\tilde{x}=\frac{x}{L}\quad,\quad\tilde{t}=\frac{t}{t^*}\quad,\quad\widetilde{\Omega}=\Omega t^* \tag{3}$$

where, $t^*=\sqrt{\frac{(\rho A)_{eq}L^4}{(EI)_{eq}}}$ is the time characteristic. Substituting the above dimensionless parameters using the characteristic time expression into the nonlinear motion's equation, and also dropping the tilde, the non-dimensional EoM is obtained as follows,

$$\frac{\partial^2 w}{\partial t^2}+\frac{\partial^4 w(x)}{\partial x^4}-\left[P+\alpha_1\int_0^1\left(\frac{\partial w}{\partial x}\right)^2dx\right]\frac{\partial^2 w}{\partial x^2}=\frac{\alpha_2\left(V_{DC}+V_s(t)\right)^2}{(1-w)^2} \tag{4}$$

$$w(0;t)=w'(0;t)=0\ ,\qquad w(1;t)=w'(1;t)=0$$

where, $\alpha_1=\frac{(EA)_{eq}d^2}{2(EI)_{eq}}$, $\alpha_2=\frac{\epsilon bL^4}{2d^3(EI)_{eq}}$ and $P=\frac{NL^2}{EI}$.

2.1.1. Static analysis

The purpose of this section is to evaluate the static displacement of the clamped-clamped microbeam under pure electrostatic DC voltage. To this aim, one should eliminate all the time-derivative terms and the non-autonomous term arising from the AC part of electrostatic actuation in Eq. (4). This results in the following static equation.

$$\frac{\partial^4 w(x)}{\partial x^4} - \left[P + \alpha_1 \int_0^1 \left(\frac{\partial w}{\partial x}\right)^2 dx\right]\frac{\partial^2 w}{\partial x^2} = \frac{\alpha_2 V_{DC}^2}{(1-w)^2} \tag{5}$$

To calculate the static deflection of the beam, multi-mode Galerkin technique is utilized, meaning that the microbeam's displacement is expanded as a linear combination of the eigenfunctions of the corresponding linear undamped system as follows:

$$w(x;t) = \sum_{i=1,3,5,\ldots}^{m} c_i \varphi_i(x) \tag{6}$$

where, φ_is are the eigenfunctions of the corresponding linear undamped system, and c_is are the constant coefficient. Introducing Eq. (6) into the nonlinear static equation, Eq. (5), and multiplying both sides of the equation by φ_i, then integrating the result over [0,1], given:

$$\sum_{i=1}^{N} c_i \int_0^1 \varphi_n \varphi_i'''' dx - P\sum_{i=1}^{N} c_i \int_0^1 \varphi_n \varphi_i'' dx - \alpha_1 \sum_{i=1}^{N}\sum_{j=1}^{N}\sum_{k=1}^{N} c_i c_j c_k \left(\int_0^1 \varphi_i' \varphi_j' dx\right)\left(\int_0^1 \varphi_n \varphi_k'' dx\right) = \alpha_2 V_{DC}^2 \sum_{i=1}^{N} \int_0^1 \frac{\varphi_n}{(1-c_i\varphi_i)^2} dx \tag{7}$$

Eq. (7) introduces a set of nonlinear algebraic equations that need to be solved numerically, so that for each specific value of the DC voltage a vector of c_i constant is computed, and consequently, the beam displacement is obtained using Eq. (6). It should be noted that the displacement-voltage behavior of the MEMS can be captured by sweeping the DC voltage value from zero up to pull-in voltage.

2.1.2. Dynamic analysis

After the bias DC voltage is statically applied to the MEMS resonator, it deflects towards the fixed substrate and settles down to its static equilibrium position. At this point, if the microbeam is excited by samll a disturbance, it starts to oscillate around this equilibrium position. Actually, the free vibration characteristics of the microbeam around the new equilibrium postion differs from those where the system is vibrating around zero equilibrium point.

To obtain the shifted natural frequencies and mode shapes of the microbeam, one needs to obtain the corresponding linear system oscillating around the new static equilibrium position (calculated in the previous section), leading to a linear eigenvalue problem.

In order to derive the linear system oscillating around the shifted static equilibrium position, we separate the total transverse displacement into two components, one defines the static displacement $w_s(x)$, and one introduces the dynamic displacement, $w_d(x;t)$, including the oscillatory information around the static position, namely:

$$w(x;t) = w_s(x) + w_d(x;t) \tag{8}$$

Substituting Eq. (8) into the non-dimensional motion's equation presented by Eq. (4), given,

$$\ddot{w}_d + w_s'''' + w_d'''' - \alpha_1 (w_s'' + w_d'') \int_0^1 \left({w_s'}^2 + {w_d'}^2 + 2w_s'w_d' \right) dx = F_{ele}(w) = \frac{\alpha_2 V_{DC}^2}{(1-w_s-w_d)^2} \quad (9)$$

Expanding the integral terms, and multiplying them by their coefficients, yields,

$$\ddot{w}_d + w_s'''' + w_d'''' - \alpha_1 w_s'' \int_0^1 {w_s'}^2 \, dx - \alpha_1 w_s'' \int_0^1 \left({w_d'}^2 + 2w_s'w_d' \right) dx - \alpha_1 w_d'' \int_0^1 {w_s'}^2 \, dx - \alpha_1 w_d'' \int_0^1 \left({w_d'}^2 + 2w_s'w_d' \right) dx = F_{ele}(w) = \frac{\alpha_2 V_{DC}^2}{(1-w_s-w_d)^2} \quad (10)$$

Then, we need to linearize the electrostatoc force in the right-hand side of the equation using Taylor series expansion.

$$F_{ele}(w) = \frac{\alpha_2 V_{DC}^2}{(1-w)^2} = \alpha_2 V_{DC}^2 \left(\frac{1}{(1-w_s)^2} + \frac{2w_d}{(1-w_s)^3} + H.O.T \right) \quad (11)$$

Introducing the linear expression for the electrostatic force into Eq. (10) results in the following form.

$$\ddot{w}_d + w_s'''' + w_d'''' - \alpha_1 w_s'' \int_0^1 {w_s'}^2 \, dx - \alpha_1 w_s'' \int_0^1 \left({w_d'}^2 + 2w_s'w_d' \right) dx - \alpha_1 w_d'' \int_0^1 {w_s'}^2 \, dx - \alpha_1 w_d'' \int_0^1 \left({w_d'}^2 + 2w_s'w_d' \right) dx = \frac{\alpha_2 V_{DC}^2}{(1-w_s)^2} + \frac{2\alpha_2 V_{DC}^2 w_d}{(1-w_s)^3} \quad (12)$$

Moreover, from the static equation, there is a balance on $w_s(x)$ between the second and fourth terms appearing in the left-hand side of Eq. (12), and the first term emerged in the right-hand side of the same equation. Finally, the non-dimensional motion's equation governing the dynamics of w_d displacement is obtained in the form of,

$$\ddot{w}_d + w_d'''' - \alpha_1 w_s'' \int_0^1 \left({w_d'}^2 + 2w_s'w_d' \right) dx - \alpha_1 w_d'' \int_0^1 {w_s'}^2 \, dx - \alpha_1 w_d'' \int_0^1 \left({w_d'}^2 + 2w_s'w_d' \right) dx = \frac{2\alpha_2 V_{DC}^2 w_d}{(1-w_s)^3} \quad (13)$$

To decompose the linear and nonlinear stiffness, we can separate the first integral into two parts, for better clarification.

$$\ddot{w}_d + \overbrace{w_d'''' - \alpha_1 w_s'' \int_0^1 2w_s'w_d' \, dx - \alpha_1 w_d'' \int_0^1 {w_s'}^2 \, dx}^{Linear\ stiffness}$$

$$\underbrace{- \alpha_1 w_s'' \int_0^1 {w_d'}^2 \, dx - \alpha_1 w_d'' \int_0^1 \left({w_d'}^2 + 2w_s'w_d' \right) dx}_{Nonlinear,\ \ quadratic\ and\ cubic\ stiffness} = \overbrace{\frac{2\alpha_2 V_{DC}^2 w_d}{(1-w_s)^3}}^{Linear\ stiffness} \quad (14)$$

Because the linear system contains integral terms, it is sophisticated to obtain its eigenvalue problem using the method of separation of variables. Therefore, we prefer to use the numerical approch which is based on Galerkin's technique to assess the linear natural frequencies and the corresponding mode shapes. In this method, we expand the system mode shapes in terms of the eigenfunctions of the associated linear system oscillating around zero equilibrium position. To this aim, the form of the shifted linear eigenvalue problem should be constructed through ignoring the nonlinear stiffness terms and substituting $w_d(x;t) = \psi(x)e^{i\lambda t}$ in Eq. (14), which yields:

$$-\omega^2\psi e^{i\lambda t}+\psi''''e^{i\lambda t}-\alpha_1 w_s'' e^{i\lambda t}\int_0^1 2w_s'\psi'\,dx-\alpha_1\psi'' e^{i\lambda t}\int_0^1 {w_s'}^2\,dx=\frac{2\alpha_2 V_{DC}^2\psi(x)e^{i\lambda t}}{(1-w_s)^3} \quad (15)$$

Canceling out $e^{i\lambda t}$ from both sides of Eq. (15), we have,

$$-\lambda^2\psi+\psi''''-\alpha_1 w_s''\int_0^1 2w_s'\psi'\,dx-\alpha_1\psi''\int_0^1 {w_s'}^2\,dx=\frac{2\alpha_2 V_{DC}^2\psi(x)}{(1-w_s)^3} \quad (16)$$

According to Galerkin's procedure, we can expand $\psi(x)$ as a linear combination of the eigenfunctions of the associated linear system vibrating around zero equilibrium position.

$$\psi(x)=\sum_{i=1}^{N} c_i\varphi_i(x) \quad (17)$$

Introducing this series into Eq. (16), and multiplying both sides of the equation by $\varphi_n(x)$, and then integrating over [0,1], it results in the following form.

$$-\lambda^2\sum_{i=1}^{N} c_i\int_0^1\varphi_n\varphi_i\,dx+\sum_{i=1}^{N} c_i\int_0^1\varphi_n\varphi_i''''\,dx-2\alpha_1\sum_{i=1}^{N} c_i\left(\int_0^1\varphi_n w_s''\,dx\right)\left(\int_0^1 w_s'\varphi_i'\,dx\right)$$
$$-\alpha_1\sum_{i=1}^{N} c_i\left(\int_0^1\varphi_n\varphi_i''dx\right)\left(\int_0^1 {w_s'}^2\,dx\right)=2\alpha_2 V_{DC}^2\sum_{i=1}^{N} c_i\int_0^1\frac{\varphi_n\varphi_i}{(1-w_s)^3}dx \quad (18)$$

Combining the sum, yields

$$-\lambda^2\sum_{i=1}^{N} c_i\delta_{ni}+\sum_{i=1}^{N}\begin{bmatrix}\int_0^1\varphi_n\varphi_i''''dx-2\alpha_1\left(\int_0^1\varphi_n w_s''\,dx\right)\left(\int_0^1 w_s'\varphi_i'\,dx\right)\\ -\alpha_1\left(\int_0^1\varphi_n\varphi_i''dx\right)\left(\int_0^1 {w_s'}^2\,dx\right)-2\alpha_2 V_{DC}^2\int_0^1\frac{\varphi_n\varphi_i}{(1-w_s)^3}dx\end{bmatrix}c_i=0$$
(19)

We can also write the matrix form of the above equation.

$$([\mathbf{K}]-\lambda^2[\mathbf{M}])\{\mathbf{C}\}=0 \quad (20)$$

here, the mass matrix, **M,** is the same as identity matrix, and the components of the linear stiffness matrix, **K**, are defined as follows.

$$K_{ni}=\omega_n^2\delta_{ni}-2\alpha_1\left(\int_0^1\varphi_n w_s''\,dx\right)\left(\int_0^1 w_s'\varphi_i'\,dx\right)-\alpha_1\left(\int_0^1\varphi_n\varphi_i''dx\right)\left(\int_0^1 {w_s'}^2\,dx\right)-2\alpha_2 V_{DC}^2\int_0^1\frac{\varphi_n\varphi_i}{(1-w_s)^3}dx \quad (21)$$

In order to evaluate the shifted/tuned natural frequencies, we should take the square root of the obtained eigenvalues, $\omega_n=\sqrt{\lambda_n}$. Here, ω_n is the natural frquency of the clamped-clamped beam vibrating around zero equilibrium, and δ_{ni} denotes the Dirac Delta. It is worth noting that because the beam is subjected to symmetric boundary conditions, only the symmetric modes (the first five symmetric modes) are considered in this analysis. Sweeping the residual axial force from zero to a positve maximum value within the reasonable range mentioned before, the variation trend of the fundamental natural frequecy of the microbeam is illistrated in Figure (…). However,

for compressive axial force, it is observed that the beam undergoes buckling instability while the resiual stress approaches -10.14 MPa.

2.1.3. Stress analysis

Theoretically specking, for beams under bending vibration, the maximum normal stress happens at the point where the beam curvature is maximum. First, the position of the point at which the normal stress becomes maximum should be detected, and then, the maximum von-Mises stress is obtained. By definition, the von-Mises stress can be expressed in terms of stress components as follows,

$$\sigma_v = \sqrt{\frac{(\sigma_{xx}-\sigma_{yy})^2+(\sigma_{xx}-\sigma_{zz})^2+(\sigma_{yy}-\sigma_{zz})^2+6(\sigma_{xy}^2+\sigma_{xz}^2+\sigma_{yz}^2)}{2}} \quad (22)$$

In case study, we assume that the normal stress σ_{xx} is the only non-zero component of the stress tensor developed at each particular material point of the structure, so the von-Mises expression is reduced to:

$$\sigma_v = \sigma_{xx} \quad (23)$$

The stress becomes maximum where the bending stress is maximum because the stress caused by mid-plane stretching and residual tensile/compressive load are constant along the longitudinal direction of the microbeam, for a specific displacement. Moreover, the bending stress is maximum where the curvature is maximum. The microbeam curvature that is contributed by bending, right before pull-in, is illustrated below.

$$\frac{1}{\rho(x)} = \frac{d^2 w_s}{dx^2} \approx \sum_{i=1}^{N} c_i \varphi_i''(x) \quad (24)$$

The microbeam curvature obtained from ROM is indicated below. As seen in Figure S.1, the beam curvature finds its maximum at the fixed ends, therefore, the maximum bending stress, and consequently the maximum stress happens at the supports.

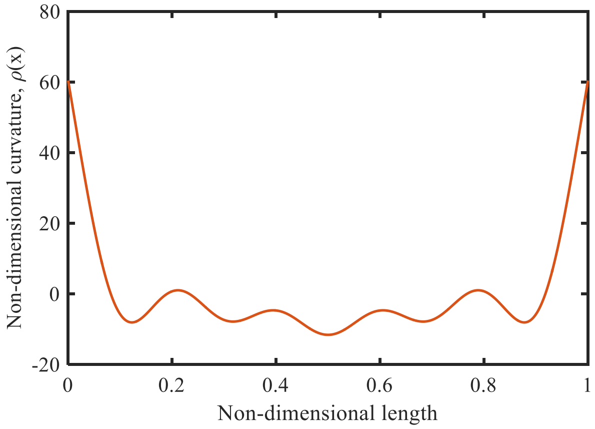

Figure S1. Non-dimensional curvature of the microbeam right before pull-in instability.

The static displacement of the beam right before pull-in is depicted in Figure S.2.

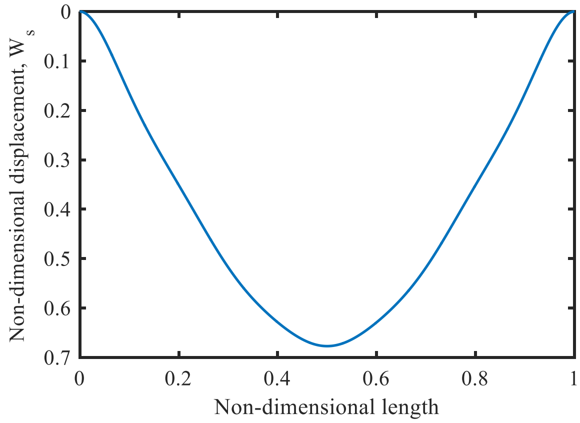

Figure S2. Non-dimensional beam displacement right before pull-in instability.

It is worth mentioning that while the beam bends downwards to the substrate, the top surface undergoes tensile stress, whereas the bottom surface experiences compressive stress. At the beam cross-section connected to the left support, the von-Mises stress is calculated for a point placed on the top surface of the beam. First, the total axial strain should be evaluated for this point, and then the total stress is obtained for the t.

$$\varepsilon_{xx}^{Tot} = -z\frac{d^2w}{dx^2} + \frac{N}{EA} + \frac{1}{2L}\int_0^L \left(\frac{\partial w}{\partial x}\right)^2 dx = -\frac{zd}{L^2}\frac{d^2\tilde{w}}{d\tilde{x}^2} + \frac{I}{AL^2}P + \frac{d^2}{2L^2}\int_0^1 \left(\frac{\partial \tilde{w}}{\partial \tilde{x}}\right)^2 d\tilde{x}$$

$$\sigma_{xx}^{Tot} = E\varepsilon_{xx}^{Tot} \quad @\ z = -\frac{h}{2}$$

3. SEM images without color

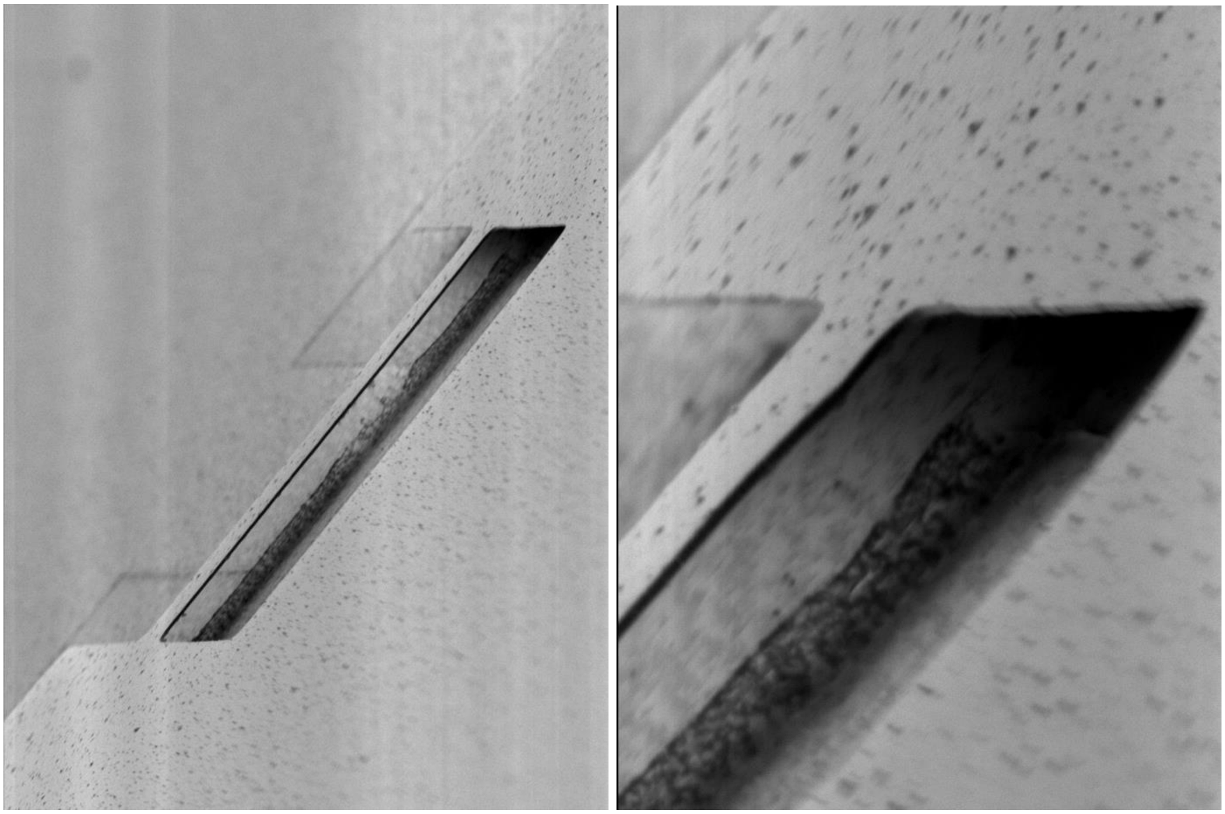

Figure S3. SEM images of fabricated resonator without color

4. Frequency sweep calculations

The data read from the oscilloscope is shown in Figure 8. Since the oscilloscope records discrete data points for each channel, input, and output signals are nx1 row vectors as, and ith element of each vector can be defined as X_i, and Y_i, respectively where both i and n are positive integers. Then, the input signal can be transformed into normalized actuation force as

$$F_i = \mathrm{X}_\mathrm{i}^2 / \max\,(X_i^2) \tag{25}$$

Then, the actuation frequency corresponding to the ith row can be found as

$$freq_i = \mathrm{f}_{start} + \frac{i}{n}(f_{stop} - f_{start}) \tag{26}$$

The velocity of the ith element can be found as

$$v_i = \mathrm{m}\,\mathrm{X}_\mathrm{i} \tag{27}$$

where m is the calibration factor and set from the LDV software as 125 mm/s/V. At this point, all the data is ready, and the rms velocity can be found as follows. First, the jth positive peak of normalized force and velocity are found by using "findpeaks()" function in MATLAB as k_j, and l_j, respectively. Then, the corresponding frequency and RMS velocity between the jth and (j+1)th peaks can be found as,

$$f_j = \frac{\sum_{i=l_j}^{l_{j+1}} freq_j}{l_{j+1} - l_j} \tag{28}$$

$$V_j = \sqrt{\frac{\sum_{i=l_j}^{l_{j+1}} v_i^2}{l_{j+1} - l_j}} \tag{29}$$

and can be plotted by plotting V_j with respect to f_j. Secondly, the phase difference between force and velocity can be found by,

$$phase_j = f_{k_j} - f_{l_j} \frac{2pi}{k_{\mathrm{j}+1} - k_j} \tag{30}$$

5. Mode shape video

The mode shape video is given supplied with “ModeShape.gif” file.
