# Supplementary figures and images for: Built-In Packaging for Single Terminal Devices
Source: Sensors (Basel). 2022 Jul 14;22(14):5264. doi: 10.3390/s22145264 (PMC9318481; doi:10.3390/s22145264)

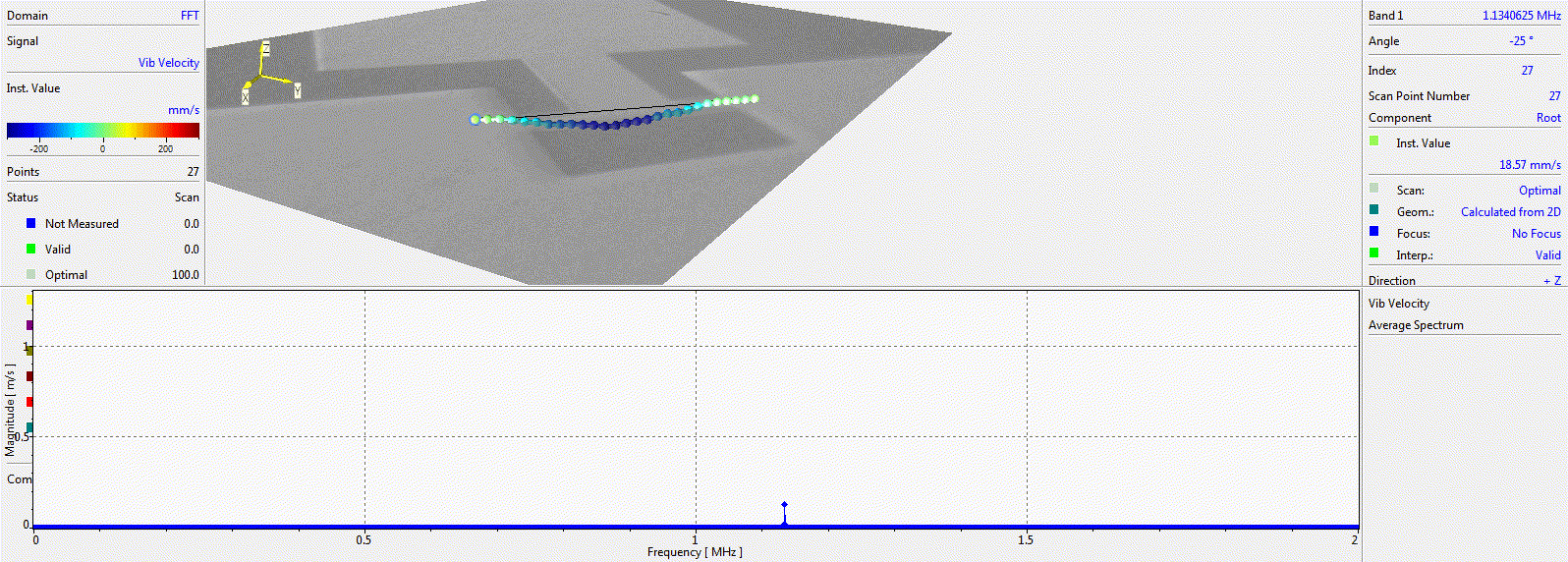

Supplement: Supplementary file 1 [file sensors-22-05264-s001.zip › ModeShape.gif]
